# Supplementary material for: Sex Disparity in the Nutrition-Related Determinants of Mild Cognitive Impairment: A Case–Control Study
Source: Nutrients. 2025 Jan 10;17(2):248. doi: 10.3390/nu17020248 (PMC11767367; doi:10.3390/nu17020248)
Supplement: Supplementary file 1 [file nutrients-17-00248-s001.zip › nutrients-3418878-supplementary.pdf]

Supplementary materials

Table S1 Examples of food items constituting the 18 food groups (from FFQ)

**Plant Food Groups Healthy**	**PDI**	**APDI**	**uPDI**
Whole grains	Positive scores	Positive scores	Reverse scores
Fruits	Positive scores	Positive scores	Reverse scores
Vegetables	Positive scores	Positive scores	Reverse scores
Nuts	Positive scores	Positive scores	Reverse scores
Legumes	Positive scores	Positive scores	Reverse scores
Vegetable oils	Positive scores	Positive scores	Reverse scores
Tea & Coffee	Positive scores	Positive scores	Reverse scores
**Less healthy**			
Fruit Juices	Positive scores	Reverse scores	Positive scores
Refined grains	Positive scores	Reverse scores	Positive scores
Potatoes	Positive scores	Reverse scores	Positive scores
Sweetened beverages	Positive scores	Reverse scores	Positive scores
Sweets & Desserts	Positive scores	Reverse scores	Positive scores
**Animal - Food Groups**			
Animal fat	Reverse scores	Reverse scores	Reverse scores
Dairy	Reverse scores	Reverse scores	Reverse scores
Egg	Reverse scores	Reverse scores	Reverse scores
Fish or Seafood	Reverse scores	Reverse scores	Reverse scores
Meat	Reverse scores	Reverse scores	Reverse scores
Misc. animal - based foods	Reverse scores	Reverse scores	Reverse scores

Table S2 Basic characteristics of elderly population aged 55 and above.

Variables		Value
Total		1086
Gender#		
	Women	572(52.6)
	Men	514(47.4)
Region#		
	Urban	555(51.2)
	Rural	530(48.9)
Age , year#		
	55~65	372（34.3）
	65~75	480（44.2)
	75~	233(21.5)
Educational level#		
	Junior school or below	928(86.5)
	High school or above	145(13.5)
Job#		
	employed or re-employ after retirement or seeking employment	167(15.4)
	Retirement or unemployed	918(84.6)
marriage#		
	married	920(84.8)
	unmarried/ divorced/widowed	165(15.2)
Live alone#		
	Yes	56(5.2)
	No	1029(94.8)
BMI（kg/m^2）#		
	＜24.0	582(55.3)
	≥24~<28	372(35.3)
	≥28	99(9.4)
Depression#		
	Yes	43(4.0)
	No	1042(96.0)
Cereal intake#, 2		
	<300 g	505(46.5)
	≥300 g	581(53.5)
Vegetables intake#		
	<150 g	533(49.1)
	≥150 g	553(50.9)
Fruit intake#		
	<50 g	652(60.0)
	≥50 g	434(40.0)
Soybean intake#		

		<38 g	710(65.4)
		≥38g	376(34.6)
Vegetable oil intake#			
		<22 g	616(56.7)
		≥22g	470(43.3)
Coffee intake#			
		No	1066(99.0)
		Yes	11（1.0）
Chocolate intake#			
		No	1047(96.3)
		Yes	40(3.7)
Strong teat intake#			
		No	894（82.2)
		Yes	193(17.8)
Nuts intake#			
		<5 g	746(68.6)
		≥5 g	341(31.4)
plant-based diet indices*		PDI	49.5±6.5
		hPDI	57.3±5.0
		uPDI	55.0±7.6
Sarcopenia related#	Sarcopenia		
		No	1019(94.7)
		Yes	57(5.3)
	low muscle mass		
		No	846(90.9)
		Yes	85(9.1)
	low muscle strength or performance		
		No low muscle strength or performance	492(46.3)
		Low muscle strength or performance	447(42.1)
		low muscle strength and performance	124(11.7)

[1] * Values are mean ± SD; # values are N (%).

[2] Cereal included rice and wheat and their product.
